# Avelumab Maintenance Therapy in Advanced Urothelial Carcinoma: Implications of Timing and Treatment Sequencing

**DOI:** 10.3390/cancers17050898

**Published:** 2025-03-06

**Authors:** Lisa Gonçalves, Helena Guedes, Ana Raquel Fortuna, Tânia Lemos, João Gramaça, Natacha Mourão, Gonçalo Cunha, Rita Pichel, Pedro Simões, Joana Alves Luís, Rita Freitas, Inês Dunões, Ana Sofia Spencer, Joana Marinho, Luís Costa

**Affiliations:** 1 Department of Oncology, Unidade Local de Saúde Santa Maria Lisboa, Avenida Professor Egas Moniz, 1649-035 Lisboa, Portugal; 2 Unidade Local de Saúde Gaia e Espinho, 4434-502 Vila Nova de Gaia, Portugal; 3 Unidade Local de Saúde Algarve, 8000-386 Faro, Portugal; 4 Unidade Local de Saúde São João, 4200-319 Porto, Portugal; 5 Unidade Local de Saúde Arco Ribeirinho, 2834-003 Barreiro, Portugal; 6 Unidade Local de Saúde Trás os Montes e Alto Douro, 5000-508 Vila Real, Portugal; 7 IPO Coimbra, 3000-075 Coimbra, Portugal; 8 Unidade Local de Saúde Santo António, 4099-001 Porto, Portugal; 9 Unidade Local de Saúde Loures Odivelas, 2674-514 Loures, Portugal; 10 Hospital dos Lusíadas, 1500-458 Lisboa, Portugal; 11 Unidade Local de Saúde Amadora/Sintra, 2720-276 Amadora, Portugal; 12 Unidade Local de Saúde Alentejo Central, 7000-811 Évora, Portugal

**Keywords:** urothelial carcinoma, avelumab, treatment sequencing, immunotherapy, chronotherapy

## Abstract

Background: Platinum-based chemotherapy (PBC) followed by avelumab maintenance is a treatment option for patients with advanced urothelial carcinoma (aUC) patients. However, the optimal treatment sequencing in the era of antibody–drug conjugates (ADCs) is yet to be determined. Recent studies suggest that the timing of immune checkpoint inhibitor (ICI) administration may impact patient outcomes, with a potential benefit from morning infusions. Methods: This retrospective study included 105 patients with aUC treated with avelumab in Portugal and intended to assess the safety and clinical outcomes (progression-free survival (PFS), overall survival (OS), and overall response rate (ORR)) and evaluate the impact of treatment timing (morning vs. afternoon) on patient outcomes. Results: The median follow-up from the start of avelumab was 17.7 months, the median PFS (mPFS) was 9.8 months (95% CI 4.9–14.7), and the median OS (mOS) was 39.5 months (95% CI 13.2–65.7). Immune-related adverse events (irAEs) were reported in 65.8% of patients, with 6.7% experiencing G3 irAEs. Among those who received a subsequent-line ADC upon disease progression (43%), the mOS from the start of avelumab was 23.1 months (95% CI 9.2–37.0). Multivariate analysis showed significant improvement in mOS with morning avelumab infusions (HR 0.35, 95% CI: 0.12–0.97, *p* = 0.042) and a trend towards improved mPFS (HR 0.43, 95% CI: 0.179–1.02, *p* = 0.055). Conclusions: This study confirms the clinical efficacy and safety of avelumab, showing improved outcomes over JAVELIN Bladder 100 and suggesting that morning infusions may offer a survival benefit in this context. Further research is needed to optimize treatment sequencing and explore the impact of infusion timing in ICI strategies.

## Simple Summary

This Portuguese real-world data study explored the effectiveness of avelumab for advanced bladder cancer and whether the timing of its administration (morning vs. afternoon) affects outcomes. Among 105 patients, avelumab delayed cancer progression for an average of 9.8 months, with a median overall survival (mOS) of 39.5 months. Long-term disease control (over 12 months) was observed in 32.4% of patients. Additionally, almost half of patients received antibody–drug conjugates after disease progression, with an mOS of 23.1 months. Notably, morning treatments led to better survival outcomes. These findings highlight the importance of optimizing treatment sequencing and timing for better patient outcomes.

## 1. Introduction

Urothelial carcinoma (UC), the predominant form of bladder cancer, poses significant therapeutic challenges, particularly in advanced stages [1]. Considering that UC is, according to Globocan 2022, the 9th most diagnosed cancer worldwide and the 13th leading cause of cancer-related deaths, more effective treatments are warranted to improve the prognosis of these patients [2].

Until recently, the standard first-line (1L) treatment for advanced urothelial carcinoma (aUC) included platinum-based chemotherapy (PBC). This regimen, containing a combination of cisplatin plus gemcitabine (CG), showed similar survival advantage when compared with methotrexate, vinblastine, doxorubicin, and cisplatin (MVAC), with a better safety profile and tolerability in advanced or metastatic bladder cancer [3].

For patients who are ineligible for cisplatin according to the Galsky criteria [4], gemcitabine plus carboplatin is an alternative. This strategy has showed to be well tolerated and as effective as other regimens, such as MCAVI (methotrexate, carboplatin, and vinblastine), with a similar overall survival (OS) and progression-free survival (PFS) in the Phase II/III EORTC 30986 trial [5].

UC is characterized by genomic instability, increased programmed cell death ligand 1 (PD-L1) protein expression, mutations in DNA damage response pathways, and a high tumour mutational burden. These characteristics are likely predictors for a favourable response to immune checkpoint inhibitors (ICIs) [6], which have been changing the treatment landscape.

The results of the JAVELIN Bladder 100 Phase III trial led to the approval of avelumab, a human anti-PD-L1 antibody, as a 1L maintenance therapy after PBC in aUC patients who did not experience disease progression, regardless of the PD-L1 status [7]. Updated results in the overall population showed an increase in OS and PFS with avelumab versus best supportive care (BSC), with a hazard ratio (HR) of 0.76 [95% CI, 0.63 to 0.91] and 0.54 [95% CI, 0.46 to 0.64], respectively [8]. In this setting, avelumab 1L maintenance treatment after PBC was the standard of care (SOC) for aUC up until recently.

In 2024, the EV-302 trial showed a clear benefit of combining the antibody–drug conjugate (ADC) enfortumab–vedotin (EV) with the ICI pembrolizumab, compared with PBC. This combination is now considered the SOC, when available and in the absence of contraindications (ESMO Cat IA, MCBS 4) [9,10]. Furthermore, in settings where EV–pembrolizumab is not available, the combination of cisplatin–gemcitabine with the ICI nivolumab may serve as a viable 1L alternative in cisplatin-eligible patients [11], and PBC followed by the ICI avelumab maintenance remains an option.

The landscape of aUC treatment is evolving rapidly. Even though the EV-302 trial established a new standard of care, economic and availability constraints may limit the access to this treatment in many countries. With a multitude of drugs now approved for aUC, the real-world impact of different treatment sequencing patterns has not been evaluated and compiled, which highlights the need for further research into optimizing treatment strategies.

Recent studies have explored the factors that may influence treatment outcomes, such as the timing of immunotherapy administration [12]. Evidence from studies in the lung [13], oesophagus [14], and kidney [15] suggest that morning infusions of immunotherapy could improve OS and PFS [16]. In fact, we have previously reported results from patients with metastatic melanoma receiving immunotherapy, showing that receiving more than 75% of the infusions in the afternoon was associated with worse OS outcomes (14.9 vs. 38.1 months; HR 0.45 (CI 0.23–0.86); *p* < 0.01) [17].

Chrono-immunotherapy is an emerging and promising field, with circadian oscillations observed in immune cell numbers as well as the expression of immunotherapy targets [12]. In a mouse melanoma model, it was recently demonstrated that dendritic cells and CD8+ T cells exhibit circadian-regulated anti-tumour activity, contributing to the control of melanoma progression and response to immunotherapy [18]. Wang et al. showed that both the quantity and quality of tumour-infiltrating lymphocytes (TILs), namely CD8+ T cells, exhibit circadian oscillations. These oscillations are dependent on the circadian clock of endothelial cells in the tumour microenvironment, which influences both the endogenous circadian clock of leukocytes and rhythmic leukocyte infiltration [19].

Despite these findings and the available evidence in distinct tumours, the underlying immune mechanisms governing chrono-immunotherapy remain unclear. However, it has been demonstrated that in mouse melanoma models, the variation in treatment efficiency with timing of administration aligns with increased tumour-associated macrophages and rhythmic expression of the Pdcd1 gene Pdcd1 (encoding PD-1) [20]. Human cancer data also revealed diurnal variations in PD-1 and PD-L1 (CD274) expression, peaking during the resting phase [21]. Disrupted circadian rhythms are linked to higher PD-1/PD-L1 levels and T cell exhaustion, suggesting that circadian regulation of immune checkpoints may impact cancer progression and the response to therapy, supporting the potential of chrono-immunotherapy.

The objective of this study was to analyse real-world data on the efficacy and safety of avelumab maintenance in the treatment of advanced or metastatic UC. Additionally, we aimed to assess subsequent treatment lines and characterize their benefit and examined the potential impact of the infusion’s timing (morning vs. afternoon) on patient outcomes.

## 2. Materials and Methods

### 2.1. Study Design and Population

This was a multicentric, retrospective cohort study in 13 Portuguese centres, including patients with aUC who received maintenance immunotherapy with avelumab. The study was conducted between September 2021 and September 2023 and patients were followed until the date of death or the last medical record entry (30 April 2024).

All patients were 18 years of age or older and were required to have (i) completed at least three cycles of 1L PBC (cisplatin–gemcitabine, carboplatin–gemcitabine or dose-dense methotrexate, vinblastine, doxorubicin and cisplatin (ddMVAC)); (ii) stable disease (SD), partial response (PR), or complete response (CR) as the best response; and (iii) performed at least one cycle of maintenance therapy with intravenous avelumab (800 mg), every two weeks. Patients were excluded if the relevant clinical data were missing.

Data regarding demographic and clinical-pathological characteristics, infusion reactions, immune-related adverse events (irAEs), imagiological responses, the timing of administration of each immunotherapy cycle, and subsequent therapeutic lines were retrieved from the patients’ electronic medical records. AEs were categorized according to the Common Terminology Criteria for Adverse Events (CTCAE Version 5.0) [22].

Disease progression, stable disease, and partial and complete response were determined either based on RECIST 1.1 (Response Evaluation Criteria in Solid Tumours) criteria [23] or on clinical assessments. An objective response was determined as having either a partial or complete response.

Avelumab’s infusion times were retrieved from patients’ medical records and dichotomized into morning (8 a.m.–2 p.m.) and afternoon (2 p.m.–8 p.m.). Patients were grouped according to the ratio of treatments received in the afternoon, as described previously [17]. The morning group included all patients who received less than 75% of infusions after 2 p.m., and the afternoon group included all patients who received at least 75% of infusions after 2 p.m.

### 2.2. Statistical Analysis

Mann–Whitney U, Pearson’s chi-square, and Fisher’s exact tests were used to assess differences between the morning and afternoon groups.

Time-to-event outcomes were estimated using the Kaplan–Meier method and tested using Cox regression models. A multivariate analysis was performed, accounting for potential prognosis confounders (age, sex, Eastern Cooperative Oncology Group Performance Status (ECOG PS), tumour grade, and presence of visceral metastases). Lesions such as lung, liver, and peritoneum metastases were considered visceral metastases; lymph nodes, skin, and bone lesions were considered non-visceral metastases. A 95% confidence interval (CI) was considered for all tests. OS and PFS were estimated from the start of avelumab’s maintenance.

Categorical variables are presented as frequencies and percentages, and continuous variables as medians with the interquartile range (IQR). Statistical analysis was performed using SPSS statistical software, version 29.0.

Stankey charts were created with https://sankeymatic.com/build/ (accessed on 4 November 2024).

## 3. Results

### 3.1. Patients’ Characteristics

A total of 105 patients from 13 centres were included; most were men (78.1%, n = 82), with a median age of 70 years (range 45 to 87, IQR 64 to 75), and 95.2% (n = 100) were fit, defined by an ECOG PS of 0 or 1. Over one-third of the patients had never smoked (36.2%, n = 38) and the majority were former (32.4%, n = 34) or active smokers (28.6%, n = 30) (Table 1).

Most patients presented with bladder tumours (72.4%, n = 76). The large majority were at Stage IV at diagnosis (75.2%, n = 79); the remaining patients presented with initial earlier stages (Stage III: (2.9%, n = 3), Stage II (13.3%, n = 14), and Stage I (8.6%, n = 9)) and, after local treatment, progressed later to Stage IV. All patients had urothelial carcinoma, with one patient with squamous differentiation. The large majority were high-grade urothelial carcinoma (89.5%, n = 94), and the remaining were low-grade (10.5%, n = 11). Programmed death ligand 1 (PD-L1) expression was tested in 28.6% of patients (n = 30); 17 patients had a positive PD-L1 combined positive score (CPS ≥ 10) and the remaining 13 had PD-L1 with a CPS less than 10.

Most patients (53.3%, n = 56) did not have visceral metastases, and the main metastatic sites were lymph nodes (65.4%, n = 68), lung (35.2%, n = 37), liver (14.3%, n = 15), and bone (14.3%, n = 15) (Table 2).

### 3.2. First-Line (1L) Treatment

Regarding PBC, most patients were treated with cisplatin plus gemcitabine (54.3%, n = 57). As for the total number of PCB cycles, the majority completed four to six cycles: 44.8% (n = 47) had four cycles, 18.1% (n = 19) had five cycles, and 33.3% (n = 35) had six cycles. Most patients presented with a partial response after PBC (54.3%, n = 57), 8.6% had CR (n = 9), and 32.3% had SD (n = 34). Data regarding the response to PBC were missing in five patients (Table 3). The median time from the last cycle of PBC to the start of maintenance therapy with avelumab was 7 weeks (range: 1 to 30 weeks).

Concerning the time between the last PBC cycle and the first avelumab infusion, the majority presented a 4- to 10-week interval, but 15.2% of patients (n = 16) waited more than 10 weeks to start maintenance therapy, and 11.4% (n = 12) had less than a 4-week window period.

The median follow-up time from the start of avelumab was 17.7 months (range: 0.5 to 41.6).

### 3.3. Safety Data

Some irAEs were reported in 65.8% (n = 68), and the majority were G1/G2. The most commonly reported irAEs were asthenia (n = 31), pruritus (n = 19), anorexia (n = 16), rash (n = 14), thyroid disfunction (n = 14), and nausea (n = 11). Infusional reactions were described in seven patients (6.7%), with one patient experiencing G4 respiratory acidosis and stridor, following the first cycle. Grade 3 irAEs were described in 6.7% of patients (2.9% rash; 2.9% infusion reactions; fever, colitis, encephalitis and nephritis, 1% each) (Table 4); nine patients (8.6%) discontinued avelumab due to irAEs.

**Figure 1:**
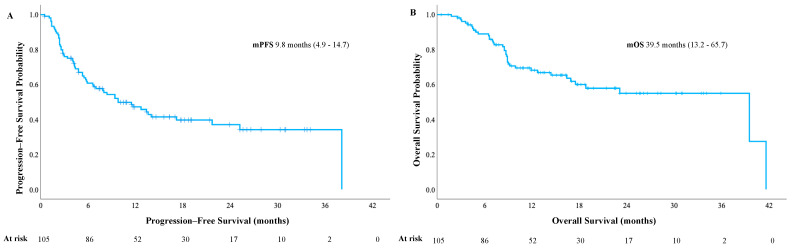
Progression–free survival (**A**) and overall survival data (**B**).

The median PFS (mPFS) was 9.8 months (95% CI, 4.9–14.7), and the median OS (mOS) was 39.5 months (95% CI, 13.2–65.7) (Figure 1A and Figure 1B, respectively). Visceral disease was associated with both lower mPFS (6.7 vs. 17.2 months, *p* = 0.034) and lower mOS (16.4 vs. 39.6 months, *p* = 0.017).

No significant differences were observed for mOS or mPFS between male and female populations, upper or lower tract disease, or number of PBC cycles.

The objective response rate (ORR) was 25.7%, and the disease control rate was 69.5%: 8.6% (n = 9) presented CR and 17.1% (n = 18) PR, and 43.8% (n = 46) had SD and 25.7% (n = 27) presented with disease progression at the first computed tomography (CT) evaluation.

Patients older than 75 years of age made up 28.6% of this cohort. The survival benefit from avelumab maintenance therapy was not significantly different between older and younger patients (mPFS *p* = 0.214; mOS *p* = 0.230).

Long-term disease control (defined as having the disease controlled for a period exceeding 12 months) was observed in 32.4% of the patients. No statistically significant differences were observed between the demographic characteristics of these long-term responders and the remaining patients from this cohort. Regarding tumour characteristics and disease burden, long-term disease control was associated with positive PD-L1 expression (CPS > 10) (*p* = 0.031); no differences were observed in tumour location or the presence of visceral metastases. G3 irAEs were also correlated with long-term disease control (*p* = 0.022).

The reasons for treatment discontinuation were disease progression (48.6%, n = 46), death (2.9%, n = 3), and irAEs (8.6%, n = 9).

### 3.4. Subsequent Therapeutic Lines

**Figure 2:**
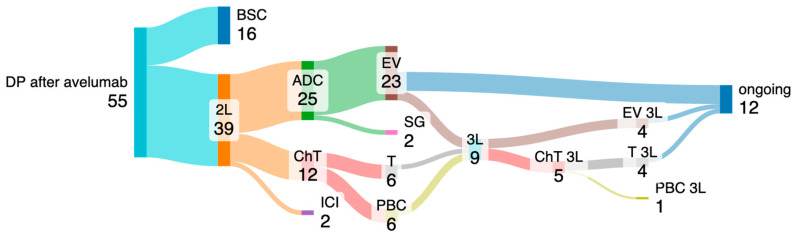
Subsequent treatment after progression under avelumab. ADC, antibody–drug conjugate; BSC, best supportive care; ChT, chemotherapy; DP, disease progression; EV, enfortumab–vedotin; ICI, immune checkpoint inhibitor; PBC, platin-based chemotherapy; SG, sacituzumab govitecan; T, paclitaxel.

In this cohort, 58 patients experienced disease progression or death during or after avelumab maintenance therapy. Upon disease progression, 67% (n = 39) of the patients were offered a subsequent therapeutic line: 25 patients (43%) were treated with an ADC (23 with EV and 2 with sacituzumab–govitecan), 2 with an anti-PD-1 antibody, and 12 with chemotherapy (ChT). After subsequent progression, nine patients received another line of therapy: five were treated with ChT and four were proposed to receive EV (Figure 2).

Among those who received a second-line (2L) or later ADC (n = 29), the mOS from the start of avelumab was 23.1 months (95% CI 9.2–37.0).

**Figure 3:**
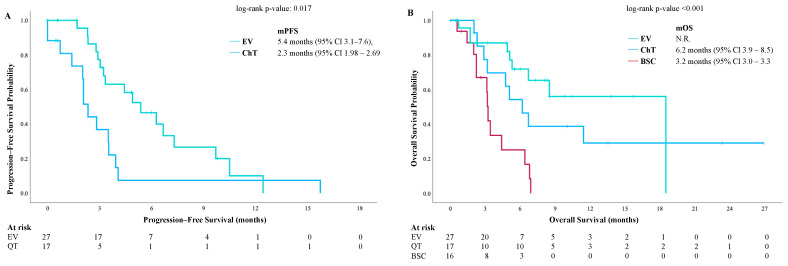
Outcomes according to subsequent lines of therapy after avelumab. Progression–free survival (**A**) and overall survival data (**B**). BSC, best supportive care; CI, confidence interval; ChT, chemotherapy; EV, enfortumab–vedotin; N.R., not reached; mOS, median overall survival; mPFS, median progression-free survival.

The mPFS for patients who received EV as a subsequent line of therapy was 5.4 months (95% CI 3.1–7.6) and 2.3 months (95% CI 1.98–2.69) for those who received ChT (*p* = 0.017) (Figure 3A). The mOS for patients receiving EV was not reached, with a 12-month OS rate of 56% (median follow-up of 6 months). In contrast, the mOS for those who received ChT was 6.2 months (95% CI 3.9–8.5), and patients who remained in BSC after progressing on avelumab had an mOS of 3.2 months (95% CI 3.0–3.3; *p* < 0.001) (Figure 3B).

### 3.5. Effect of the Time of Day of Infusion in aUC Patients Under Avelumab

The aUC patients under avelumab completed 2183 avelumab infusions. Most patients (n = 94, 89.5%) were allocated to the morning group (<75% of infusions in the afternoon) and 11 patients (10.5%) were included in the afternoon group (≥75% of infusions in the afternoon).

No bias was observed in the treatments’ allocation into morning (8 a.m.–2 p.m.) or afternoon (2 p.m.–8 p.m.) groups according to demographic characteristics (age, sex, or ECOG PS), tumour characteristics, or disease burden (Table 5).

All grades of immune-related toxicities were more frequent in the afternoon group, even though not statistically significantly (*p* = 0.055) (Table 6).

The median number of cycles of avelumab was 15 in the morning group (IQ 7–33) and 12 in the afternoon group (IQ 4–20).

The disease control rate at the first CT evaluation was 71.3% in the morning group and 54.5% in the afternoon group, with an ORR of 26.6% and 18.2%, respectively.

**Figure 4:**
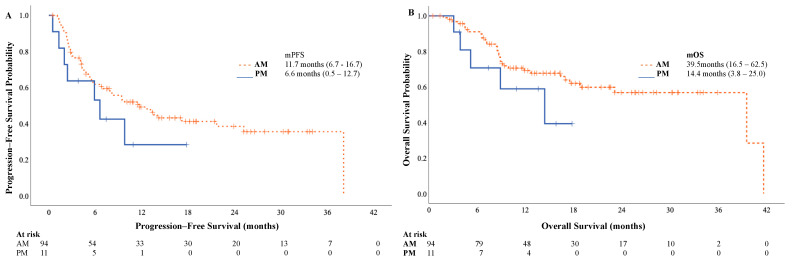
Kaplan–Meier curves of progression-free and overall survival by AM/PM treatments. (**A**) progression-free survival; (**B**) overall survival. AM (morning) group: <75% of infusions after 2 p.m.; PM (afternoon) group: ≥75% of infusions after 2 p.m.; NR, not reached.

**Figure 5:**
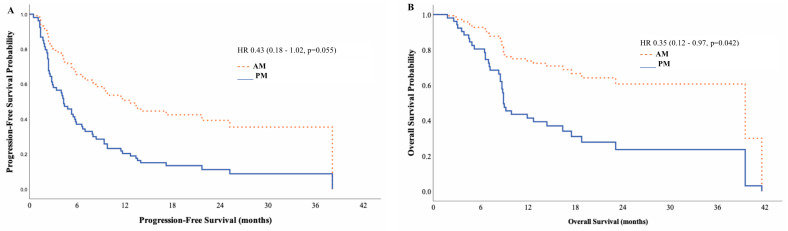
Multivariate analysis survival curves for progression-free and overall survival hazard ratio by AM/PM treatments. (**A**) Progression-free survival; (**B**) overall survival. AM (morning) group: <75% of infusions after 2 p.m.; PM (afternoon) group: ≥75% of infusions after 2 p.m.

Univariate survival analysis showed a non-statistically significant trend for an increase in mPFS and mOS in the morning group (mPFS: 11.7 vs. 6.6 months, *p* = 0.215, HR 0.61 [95% CI: 0.27–1.34]; mOS 39.5 vs. 14.4 months, *p* = 0.189; HR 0.53 [95% CI: 0.21–1.38]) (Figure 4). After performing a multivariate analysis adjusted for confounding variables (age, sex, ECOG PS, tumour grade, and the presence of visceral metastases), we found a statistically significant improvement in mOS (and a non-statistically significant trend in mPFS) between the morning and afternoon administrations (mPFS HR 0.43, 95% CI: 0.179–1.02, *p* = 0.055; mOS HR 0.35, 95% CI: 0.12–0.97, *p* = 0.042) (Figure 5).

## 4. Discussion

This multicentric study aimed to determine the characteristics of a cohort of aUC patients treated with avelumab maintenance therapy after PBC. In addition, we intended to evaluate the real-world PFS and OS benefits of this treatment sequence, in light of the original trial, JAVELIN Bladder100.

The characteristics of the patient characteristics in this real-world study were comparable with those observed in the original trial: most patients were men (78.1% vs. 77.2% in the trial) and had an ECOG PS of 0 or 1; 36.2% were non-smokers (vs. 31.3% in the trial). In terms of disease characterization, a significant proportion of this cohort had lower urinary tract tumours (72.4% compared with 73.3% in the trial). Notably, the incidence of visceral disease was slightly lower in this study cohort than in the trial population (46.7% vs. 54.6%). Additionally, as PD-L1 testing is not mandatory for avelumab maintenance therapy, it was performed in only 28.6% of the patients, a markedly lower rate than the 89.7% reported in the trial, though it aligns with other real-world data on aUC [24]. Regarding PBC, the treatment patterns reported herein were identical to those reported in the JAVELIN Bladder 100; approximately half of the patients were treated with cisplatin plus gemcitabine (54.2% vs. 52.3%) and about one-third received four cycles of PBC (33.3% in this cohort vs. 35.9% in the trial). Both studies reported that most patients had partial or complete responses after PBC, with response rates of 62.9% and 72.3%, respectively. Taken together, these data showed that the real-world aUC population under avelumab maintenance therapy closely aligns with that observed in the trial and in other real-world studies [7,25,26].

Regarding patient outcomes, to our knowledge, the mOS of 39.5 months reported in our study is the longest ever reported for patients receiving PBC followed by avelumab maintenance. This may be related to the lower proportion of patients with visceral disease compared with both the trial and other real-world analyses (46.7% vs. 54.6–81.5%) [7,25,26], as visceral disease is known to correlate with poorer prognosis. In a post hoc analysis of the JAVELIN Bladder 100 trial, OS and PFS were prolonged in subgroups of patients with a low tumour burden (non-visceral metastasis median OS, 31.4 months; lymph node disease only mOS, 31.9 months) [27].

The observation that 15.2% of patients had an interval of more than 10 weeks between the last cycle of PBC and the initiation of maintenance therapy suggests that, in some cases, there may have been an initial exclusion of early progressors—those who experienced disease progression while waiting for treatment.

Importantly, the patients in this cohort had different subsequent therapeutic lines from those of the JAVELIN Bladder 100. In our study, most patients who progressed on avelumab received an ADC as a subsequent line of treatment, in contrast to the trial population, where most patients were offered ChT [8].

Considering aUC patients who progressed with PBC and ICIs, data from the EV-301 Phase III trial showed a benefit of EV, an ADC directed to nectin-4 (a cell-adhesion molecule expressed in UC) [28]. Also in this population, the Phase III TROPiCS-04 trial showed a numerical improvement in OS favouring sacituzumab govitecan (an antibody–drug conjugate targeting the trophoblast cell surface antigen-2, which is overexpressed in UC), suggesting improvements in PFS and ORR, although the primary endpoint for OS was not achieved [29]. The sequential use of maintenance therapy with avelumab followed by ADC may have contributed to the better outcomes observed in this study, highlighting the significance of real-world data in understanding how therapeutic sequencing can affect patient outcomes.

Additionally, recent data regarding the impact of the timing of immunotherapy on the outcomes of patients with various solid tumours further emphasize the need to consider these factors in treatment strategies [13,15,16,17,24,30,31,32,33,34,35,36,37]. Our exploratory analysis examined the impact of the time of day of avelumab infusions on OS and PFS. After multivariate analysis, our data indicated that patients experienced better outcomes (mPFS and mOS) when less than 75% of the infusions are performed after 2 p.m. (morning group). Our data are in line with data from Ortega et al., who showed, in a small aUC cohort, that earlier infusions of anti-PD-1 or anti-PD-L1 agents in the first or subsequent lines are associated with significantly better outcomes [24].

This study is the first to specifically report improved outcomes for aUC patients receiving avelumab infusions in the morning and also includes a larger cohort compared with that of Ortega et al., whose study involved a smaller patient population and did not exclusively focus on a single ICI. However, our study had some limitations, namely its retrospective nature, which may add potential selection bias, and the very small number of patients included in the afternoon group (n = 11, 10.5%). Still, this is the largest series in urothelial cancer focusing on chrono-immunotherapy and the only one with only one immune checkpoint inhibitor drug. The mechanisms that underlie these differences are still not fully understood, although emerging data suggest a role of oscillating tumour infiltration of immune cells dependent on endothelial circadian clock [19]. This highlights the need for prospective translational studies.

## 5. Conclusions

Even though the new standard of care for aUC is the combination of ICI/ADC (pembrolizumab plus EV), in many countries, the availability of this combination is still limited. Maintenance therapy with avelumab after PBC is an established treatment option with manageable toxicities. Of note, ADCs, such as EV, as a second therapeutic line were not the standard of care at the time of the JAVELIN Bladder 100 trial, highlighting the importance of real-world data.

Overall, our data appear to be consistent with the findings from the JAVELIN Bladder 100 trial and recent real-world studies, showing favourable outcomes, with an mPFS of 9.8 months and an mOS of 39.5 months, which exceeded the survival rates reported either in the trial or in any study. Taken together, the data evidenced the magnitude of the true benefit of sequencing PBC, maintenance immunotherapy, and ADC upon disease progression.

In line with a growing body of evidence focusing on the impact of immunotherapy’s time of day of administration, our study also offers novel insights into the influence of treatment timing on survival outcomes. Although our observation requires further validation in studies with more robust designs and larger cohorts, it aligns with emerging evidence in other tumour types, suggesting that immunotherapy may be more effective when administered earlier in the day. The potential to improve outcomes by simply altering the time of administration is remarkable; however, our data underscore the need for prospective studies with a translational approach.

These findings reinforce the value of avelumab maintenance therapy in aUC, particularly in the continuum of care alongside with ADC after progression, and highlight the potential role of treatment timing as an important factor for optimizing therapeutic outcomes. 

## Figures and Tables

**Table 1:** Patients’ demographics.

Demographic Characteristics	n = 105 (N = 28)
**Age, yr**	
Median (IQR)	70 (64–75)
**Sex, n (%)**	
Male	82 (78.1)
Female	23 (21.9)
**ECOG PS, n (%)**	
0	58 (55.2)
1	42 (40.0)
2	5 (4.8)
**Smoker status, n (%)**	
Never	38 (36.1)
Former	34 (32.4)
Active	30 (28.6)
Unknown	3 (2.9)

ECOG PS, Eastern Cooperative Oncology Group Performance Status; IQR, interquartile range; n, number, yr, years.

**Table 2:** Cohort tumour characterization.

Tumour Characteristics	n (%)
**Site of primary tumour**	
Upper urinary tract	29 (27.6)
Lower urinary tract	76 (72.4)
**Site of baseline metastases**	
Visceral site	49 (46.7)
Non-visceral site	56 (53.3)
**PD-L1 status**	
Positive	13 (12.4)
Negative	17 (16.2)
Unknown	75 (71.4)

n, number; PD-L1 programmed death ligand 1.

**Table 3:** Characterization of first-line (1L) chemotherapy.

1L Chemotherapy	n (%)
Gemcitabine plus cisplatin	57 (54.3)
Gemcitabine plus carboplatin	45 (42.9)
ddMVAC	2 (1.9)
Not reported	1 (0.9)
**Number of cycles**	
3	2 (1.9)
4	47 (44.8)
5	19 (18.1)
6	35 (33.3)
≥7	2 (1.9)
**Best response to 1L chemotherapy**	
Complete response	9 (8.6)
Partial response	57 (54.3)
Stable disease	34 (32.3)
Not reported	5 (4.8)

ddMVAC: dose-dense methotrexate, vinblastine, doxorubicin, and cisplatin; n: number.

**Table 4:** Immune-related adverse events during avelumab maintenance therapy.

Immune-Related Adverse Events	Any Grade n (%)	G1/G2 n (%)	G3/G4 n (%)
Any adverse event	68 (65.8)	65 (61.9)	7 (6.7)
Asthenia	31 (29.5)	31 (29.5)	0 (0.0)
Pruritus	19 (18.1)	19 (18.1)	0 (0.0)
Anorexia	16 (15.2)	16 (15.2)	0 (0.0)
Rash	14 (13.3)	11 (10.5)	3 (2.9)
Nausea	11 (10.5)	11 (10.5)	0 (0.0)
Thyroid disfunction	14 (13.3)	14 (13.3)	0 (0.0)
Hepatitis	8 (7.6)	8 (7.6)	0 (0.0)
Diarrhoea	7 (6.7)	6 (5.7)	1 (1.0)
Infusional reactions	7 (6.7)	4 (3.8)	3 (2.9)
Fever	6 (5.7)	5 (4.8)	1 (1.0)
Hematologic alterations	6 (5.7)	6 (5.7)	0 (0.0)
Myalgias/arthralgias	5 (4.8)	5 (4.8)	0 (0.0)
Nephritis	2 (1.9)	1 (1.0)	1 (1.0)
Colitis	2 (1.9)	1 (1.0)	1 (1.0)
Headache	1 (1.0)	1 (1.0)	0 (0.0)
Encephalitis	1 (1.0)	0 (0.0)	1 (1.0)
Pneumonitis	1 (1.0)	1 (1.0)	0 (0.0)
Vitiligo	1 (1.0)	1 (1.0)	0 (0.0)

**Table 5:** Baseline characteristics of the total study cohort and according to morning vs. afternoon groups.

	Morning Group ^(a)^ (n = 94)	Afternoon Group ^(b)^ (n = 11)	*p*-Value
**Age, yr**			0.498
Median	70	71	
Range	45–87	60–80	
**Sex, n (%)**			0.277
Male	72 (76.6)	10 (90.9)	
Female	22 (23.4)	1 (9.1)	
**ECOG PS, n (%)**			0.269
0	50 (53.2)	8 (72.7)	
1	40 (42.6)	2 (18.2)	
2	4 (4.2)	1 (9.1)	
**Site of primary tumour, n (%)**			0.146
Upper urinary tract	28 (29.8)	1 (9.1)	
Lower urinary tract	66 (70.2)	10 (90.9)	
**Site of baseline metastasis, n (%)**			0.173
Visceral site	43 (45.7)	3 (27.3)	
Non-visceral site	48 (54.3)	8 (72.7)	

^(a)^ The morning group was defined as patients with <75% of infusions after 2 p.m. ^(b)^ The afternoon group was defined as patients with ≥75% of infusions after 2 p.m. ECOG PS, Eastern Cooperative Oncology Group Performance Status; irAEs, immune-related adverse events; n, number; yr, years.

**Table 6:** Immune-related adverse events in the morning and afternoon groups.

	Morning Group ^(a)^ (n = 94)	Afternoon Group ^(b)^ (n = 11)	*p*-Value
**All grades of irAE**			0.055
Present	36 (38.3)	10 (90.9)	
Absent	58 (61.7)	1 (9.1)	
**Grade 3 irAEs**			0.106
Present	5 (5.3)	2 (18.2)	
Absent	89 (94.7)	9 (81.8)	

^(a)^ The morning group was defined as patients with <75% of infusions after 2 p.m. ^(b)^ The afternoon group was defined as patients with ≥75% of infusions after 2 p.m. AE, adverse event; irAEs, immune-related adverse events.

## Data Availability

The data presented in this study are available on request from the corresponding authors. The data are not publicly available due to ethical restrictions.

## References

[1] Zhang Y., Rumgay H., Li M., Yu H., Pan H., Ni J. (2023). The global landscape of bladder cancer incidence and mortality in 2020 and projections to 2040. J. Glob. Health.

[2] Cancer TODAY|IARC. https://gco.iarc.who.int.

[3] von der Maase H., Hansen S.W., Roberts J.T., Dogliotti L., Oliver T., Moore M.J., Bodrogi I., Albers P., Knuth A., Lippert C.M. (2000). Gemcitabine and cisplatin versus methotrexate, vinblastine, doxorubicin, and cisplatin in advanced or metastatic bladder cancer: Results of a large, randomized, multinational, multicenter, phase III study. J. Clin. Oncol..

[4] Galsky M.D., Hahn N.M., Rosenberg J., Sonpavde G., Hutson T., Oh W.K., Dreicer R., Vogelzang N., Sternberg C.N., Bajorin D.F. (2011). Treatment of patients with metastatic urothelial cancer “unfit” for Cisplatin-based chemotherapy. J. Clin. Oncol..

[5] De Santis M., Bellmunt J., Mead G., Kerst J.M., Leahy M., Maroto P., Gil T., Marreaud S., Daugaard G., Skoneczna I. (2012). Randomized phase II/III trial assessing gemcitabine/carboplatin and methotrexate/carboplatin/vinblastine in patients with advanced urothelial cancer who are unfit for cisplatin-based chemotherapy: EORTC study 30986. J. Clin. Oncol..

[6] Bakaloudi D.R., Talukder R., Makrakis D., Diamantopoulos L., Enright T., Leary J.B., Patgunarajah U., Thomas V.M., Swami U., Agarwal N. (2024). Association of Tumor Mutational Burden and Microsatellite Instability With Response and Outcomes in Patients With Urothelial Carcinoma Treated With Immune Checkpoint Inhibitor. Clin. Genitourin. Cancer.

[7] Powles T., Park S.H., Voog E., Caserta C., Valderrama B.P., Gurney H., Kalofonos H., Radulovic S., Demey W., Ullen A. (2020). Avelumab Maintenance Therapy for Advanced or Metastatic Urothelial Carcinoma. N. Engl. J. Med..

[8] Powles T., Park S.H., Caserta C., Valderrama B.P., Gurney H., Ullen A., Loriot Y., Sridhar S.S., Sternberg C.N., Bellmunt J. (2023). Avelumab First-Line Maintenance for Advanced Urothelial Carcinoma: Results From the JAVELIN Bladder 100 Trial After ≥2 Years of Follow-Up. J. Clin. Oncol..

[9] Powles T., Bellmunt J., Comperat E., De Santis M., Huddart R., Loriot Y., Necchi A., Valderrama B.P., Ravaud A., Shariat S.F. (2024). ESMO Clinical Practice Guideline interim update on first-line therapy in advanced urothelial carcinoma. Ann. Oncol..

[10] Powles T., Valderrama B.P., Gupta S., Bedke J., Kikuchi E., Hoffman-Censits J., Iyer G., Vulsteke C., Park S.H., Shin S.J. (2024). Enfortumab Vedotin and Pembrolizumab in Untreated Advanced Urothelial Cancer. N. Engl. J. Med..

[11] Heijden M.S.v.d., Sonpavde G., Powles T., Necchi A., Burotto M., Schenker M., Sade J.P., Bamias A., Beuzeboc P., Bedke J. (2023). Nivolumab plus Gemcitabine–Cisplatin in Advanced Urothelial Carcinoma. N. Engl. J. Med..

[12] Pick R., Wang C., Zeng Q., Gul Z.M., Scheiermann C. (2024). Circadian Rhythms in Anticancer Immunity: Mechanisms and Treatment Opportunities. Annu. Rev. Immunol..

[13] Rousseau A., Tagliamento M., Auclin E., Aldea M., Frelaut M., Levy A., Benitez J.C., Naltet C., Lavaud P., Botticella A. (2023). Clinical outcomes by infusion timing of immune checkpoint inhibitors in patients with advanced non-small cell lung cancer. Eur. J. Cancer.

[14] Nomura M., Hosokai T., Tamaoki M., Yokoyama A., Matsumoto S., Muto M. (2023). Timing of the infusion of nivolumab for patients with recurrent or metastatic squamous cell carcinoma of the esophagus influences its efficacy. Esophagus.

[15] Patel J., Draper A., Woo Y., Dhabaan L., Patel P., Jani A., Carthon B., Master V., Kissick H., Bilen M. (2022). 848 Impact of immunotherapy time-of-day infusion on overall survival in patients with metastatic renal cell carcinoma. J. ImmunoTherapy Cancer.

[16] Landre T., Karaboue A., Buchwald Z.S., Innominato P.F., Qian D.C., Assie J.B., Chouaid C., Levi F., Duchemann B. (2024). Effect of immunotherapy-infusion time of day on survival of patients with advanced cancers: A study-level meta-analysis. ESMO Open.

[17] Gonçalves L., Goncalves D., Casanelles T.E., Guerra L.P., Menezes M.B., Branco V.D., Luís J.A., Simões F., Gramaça J., PINHO I.S. (2024). Retro TIMing: A multicentric retrospective analysis of immunotherapy timing in metastatic melanoma. J. Clin. Oncol..

[18] Wang C., Barnoud C., Cenerenti M., Sun M., Caffa I., Kizil B., Bill R., Liu Y., Pick R., Garnier L. (2023). Dendritic cells direct circadian anti-tumor immune responses. Nature.

[19] Wang C., Zeng Q., Gul Z.M., Wang S., Pick R., Cheng P., Bill R., Wu Y., Naulaerts S., Barnoud C. (2024). Circadian tumor infiltration and function of CD8^+^ T cells dictate immunotherapy efficacy. Cell.

[20] Tsuruta A., Shiiba Y., Matsunaga N., Fujimoto M., Yoshida Y., Koyanagi S., Ohdo S. (2022). Diurnal Expression of PD-1 on Tumor-Associated Macrophages Underlies the Dosing Time-Dependent Antitumor Effects of the PD-1/PD-L1 Inhibitor BMS-1 in B16/BL6 Melanoma-Bearing Mice. Mol. Cancer Res..

[21] Wu Y., Tao B., Zhang T., Fan Y., Mao R. (2019). Pan-Cancer Analysis Reveals Disrupted Circadian Clock Associates with T Cell Exhaustion. Front. Immunol..

[22] Freites-Martinez A., Santana N., Arias-Santiago S., Viera A. (2021). Using the Common Terminology Criteria for Adverse Events (CTCAE—Version 5.0) to Evaluate the Severity of Adverse Events of Anticancer Therapies. Actas Dermo-Sifiliogr..

[23] Eisenhauer E.A., Therasse P., Bogaerts J., Schwartz L.H., Sargent D., Ford R., Dancey J., Arbuck S., Gwyther S., Mooney M. (2009). New response evaluation criteria in solid tumours: Revised RECIST guideline (version 1.1). Eur. J. Cancer.

[24] Ortego I., Molina-Cerrillo J., Pinto A., Santoni M., Alonso-Gordoa T., Criado M.P.L., Gonzalez-Morales A., Grande E. (2022). Time-of-day infusion of immunotherapy in metastatic urothelial cancer (mUC): Should it be considered to improve survival outcomes?. J. Clin. Oncol..

[25] Bellmunt J., Chang J., Pavilack-Kirker M., Cappelleri J.C., Costa N., Esterberg E., Kearney M., Hitchens A., Candrilli S.D., Ajmera M. (2023). Evaluating Real-World Characteristics of Patients With Advanced Urothelial Carcinoma Eligible for Avelumab Maintenance Therapy: A Multicountry Retrospective Medical Chart Review. Clin. Genitourin. Cancer.

[26] Barthelemy P., Loriot Y., Voog E., Eymard J.C., Ravaud A., Flechon A., Jaillon C.A., Chasseray M., Lorgis V., Hilgers W. (2023). Full analysis from AVENANCE: A real-world study of avelumab first-line (1L) maintenance treatment in patients (pts) with advanced urothelial carcinoma (aUC). J. Clin. Oncol..

[27] Bellmunt J., Powles T., Park S.H., Voog E., Valderrama B.P., Gurney H., Ullén A., Loriot Y., Sridhar S.S., Tsuchiya N. (2024). Avelumab first-line maintenance (1LM) for advanced urothelial carcinoma (aUC): Long-term outcomes from JAVELIN Bladder 100 in patients (pts) with low tumor burden. J. Clin. Oncol..

[28] Powles T., Rosenberg J.E., Sonpavde G.P., Loriot Y., Duran I., Lee J.L., Matsubara N., Vulsteke C., Castellano D., Wu C. (2021). Enfortumab Vedotin in Previously Treated Advanced Urothelial Carcinoma. N. Engl. J. Med..

[29] Gilead (2024). Gilead Provides Update on Phase 3 TROPiCS-04 Study. https://www.gilead.com/news/news-details/2024/gilead-provides-update-on-phase-3-tropics-04-study.

[30] Qian D.C., Kleber T., Brammer B., Xu K.M., Switchenko J.M., Janopaul-Naylor J.R., Zhong J., Yushak M.L., Harvey R.D., Paulos C.M. (2021). Effect of immunotherapy time-of-day infusion on overall survival among patients with advanced melanoma in the USA (MEMOIR): A propensity score-matched analysis of a single-centre, longitudinal study. Lancet Oncol..

[31] Yeung C., Kartolo A., Tong J., Hopman W., Baetz T. (2023). Association of circadian timing of initial infusions of immune checkpoint inhibitors with survival in advanced melanoma. Immunotherapy.

[32] Karaboue A., Collon T., Pavese I., Bodiguel V., Cucherousset J., Zakine E., Innominato P.F., Bouchahda M., Adam R., Levi F. (2022). Time-Dependent Efficacy of Checkpoint Inhibitor Nivolumab: Results from a Pilot Study in Patients with Metastatic Non-Small-Cell Lung Cancer. Cancers.

[33] Cortellini A., Barrichello A.P.C., Alessi J.V., Ricciuti B., Vaz V.R., Newsom-Davis T., Evans J.S., Lamberti G., Pecci F., Viola P. (2022). A multicentre study of pembrolizumab time-of-day infusion patterns and clinical outcomes in non-small-cell lung cancer: Too soon to promote morning infusions. Ann. Oncol..

[34] Barrios C.H., Montella T.C., Ferreira C.G.M., Marchi P.D., Coutinho L.F., Duarte I.L., Silva M.C.e., Paes R.D., Silva G.M.C.e., Dienstmann R. (2022). Time-of-day infusion of immunotherapy may impact outcomes in advanced non-small cell lung cancer patients (NSCLC). J. Clin. Oncol..

[35] Dizman N., Govindarajan A., Zengin Z.B., Meza L.A., Tripathi N., Sayegh N., Castro D.V., Chan E.H., Lee K.O., Prajapati S.R. (2023). Association between time-of-day of the immune checkpoint inhibitor (ICI) infusion and disease outcomes among patients with metastatic renal cell carcinoma (mRCC). J. Clin. Oncol..

[36] Fernandez-Mañas L., Aguado L.G., Aversa C., Ferrer-Mileo L., Herreros M.G.d., Jiménez N., Febrer A., Vernet R., García-Esteve S., Mellado B. (2023). Does the time-of-day administration of immune checkpoint inhibitors affect efficacy in patients with metastatic renal cell carcinoma? A single-center study. J. Clin. Oncol..

[37] Vilalta A., Arasanz H., Rodriguez-Remirez M., Lopez I., Puyalto A., Lecumberri A., Baraibar I., Corral J., Gúrpide A., Perez-Gracia J.L. (2021). 967P The time of anti-PD-1 infusion improves survival outcomes by fasting conditions simulation in non-small cell lung cancer. Ann. Oncol..

